# The Role of Artificial Weathering Protocols on Abiotic and Bacterial Degradation of Polyethylene

**DOI:** 10.3390/polym17131798

**Published:** 2025-06-27

**Authors:** Pauline F. De Bigault De Cazanove, Alena Vdovchenko, Ruth S. Rose, Marina Resmini

**Affiliations:** 1 Department of Chemistry, School of Physical and Chemical Sciences, Queen Mary University of London, Mile End Road, London E1 4NS, UK; p.f.debigaultdecazanove@qmul.ac.uk (P.F.D.B.D.C.); alenavdovchjenko@gmail.com (A.V.); 2 School of Biological and Behavioural Sciences, Queen Mary University of London, Mile End Road, London E1 4NS, UK; r.s.rose@qmul.ac.uk

**Keywords:** microplastic, artificial weathering, artificial ageing, low density polyethylene, abiotic degradation, carbonyl index

## Abstract

Plastic pollution poses significant environmental challenges due to its persistence and contribution to the microplastic formation, with polyethylene being among the materials more abundantly found. Understanding how different artificial weathering protocols influence the degradation of plastics is crucial for assessing their environmental impact. This study investigates the effects of three distinct artificial weathering protocols—continuous UV-A irradiation (M_L_), cyclic UV-dark exposure (M_C[L→D]_), and sequential UV-dark phase (M_L→D_)—on the physicochemical properties of plastics, using oxo-low-density polyethylene as the model material. Surface oxidation, measured by quantification of the carbonyl index, was most pronounced under the M_C[L→D]_ protocol despite the shortest time of overall UV exposure, indicating that oxidative reactions continue during the dark phases. Vinyl group formation, however, required continuous or cyclic UV exposure, highlighting the critical role of light in this chemical process. Alterations in the surface hydrophilicity, measured by contact angle, and changes in molecular weight were quantified and found to closely link to the weathering conditions, with increased oxidations enhancing the surface hydrophilicity and the chain scission balanced by crosslinking with extended UV durations. These findings emphasize the importance of weathering protocols when trying to simulate conditions in the lab that are closer to the ones in the environment to understand plastic degradation mechanisms. Biodegradation experiments with *Rhodococcus rhodochrous* demonstrated that weathered oxo-LDPE samples with higher surface oxidation levels (ΔCI > 1) supported an increased CO_2_ production by *Rhodococcus rhodochrous*, with the M_C[L→D]_—360 h protocol yielding the highest biodegradation rates—31–43% higher than the control.

## 1. Introduction

Plastic pollution has become one of the most pressing environmental challenges of our time, with millions of tonnes of plastic waste entering ecosystems annually and affecting all geographical areas of the planet [1,2,3]. Among these, polyethylene—one of the most widely produced and discarded plastics—contributes significantly to environmental contamination due to its persistence and extensive use in single-use packaging and consumer products [4]. Over time, discarded polyethylene waste degrades into smaller fragments, forming microplastics that infiltrate both aquatic and terrestrial systems. Our recent systematic literature review identified polyethylene as one of the most common microplastic types found in meat, plant-based food, seafood, and drinking water, highlighting significant human exposure [5]. Microplastics can exhibit intrinsic toxicity or act as vectors for other contaminants, bacteria, or viruses, further amplifying their environmental and health impacts [6,7,8,9,10].

Weathering processes play a critical role in determining the environmental fate of plastics, including polyethylene, by altering their physicochemical properties over time. Abiotic degradation mechanisms, such as photooxidation, thermal degradation, and oxidative chain scission, proceed via free radical pathways and are strongly influenced by environmental conditions like UV exposure, oxygen availability, and temperature (Figure S1, Supplementary Materials). Thus, exposure to environmental factors accelerates the degradation of plastics, fragmenting them into microplastics and modifying their surface chemistry [11,12,13]. These changes influence material toxicity and interaction with biological systems and act as potential carriers for other pollutants [14,15,16].

Abiotic degradation of plastics is typically evaluated using key physicochemical parameters that describe changes in the material’s structure and surface properties. Molecular weight reduction is a critical indicator, reflecting polymer chain scission during degradation processes [17,18]. Generally, polymers with lower molecular weight tend to degrade at higher rates under both abiotic and biotic conditions, as shorter chains are more susceptible to thermal and enzymatic processes [19,20]. Surface oxidation, often measured by techniques such as infrared spectroscopy, Raman spectroscopy, and X-ray Photoelectron Spectroscopy (XPS), provides insights into chemical modifications, including the introduction of oxygen-containing functional groups [21,22,23]. Surface oxidation can lead to changes in surface hydrophobicity, commonly assessed through water contact angle measurements [24,25]. Changes in the physicochemical properties of plastics are often linked to their behaviour in biological environments, influencing processes such as eco-corona formation, biofilm formation, and susceptibility to biotic degradation [24,26]. Together, these parameters form a comprehensive framework for understanding the extent and mechanisms of abiotic plastic degradation under varying environmental conditions.

A wide range of artificial weathering protocols, which simulate environmental conditions in controlled settings, are used to study the degradation behaviour of plastics for both industrial applications and environmental research. These protocols involve exposure to UV light, temperature cycles, humidity, and mechanical abrasion to accelerate material degradation compared to real-world conditions [27,28,29]. In industrial settings, standardized methods such as ISO and ASTM are commonly employed to evaluate the biodegradability of plastics, to cite a few examples [30,31,32]. However, the protocols vary significantly in their technical parameters, making the selection of an appropriate method challenging and complicating the comparisons between materials weathered by different approaches. This variability raises important questions about how artificial weathering standards can influence the physicochemical properties and biodegradation of aged plastics. Bridging this gap is essential to understanding the changes in physicochemical properties and degradation rates that can be expected when specific weathering conditions are applied and when evaluating the toxicological and environmental impacts of plastic materials.

In this research, we studied the effects that different artificial weathering protocols had on the physicochemical properties of low-density polyethylene films containing a pro-oxidant masterbatch (oxo-LDPE) designed to accelerate degradation. Varying combinations of UV irradiation and heating, alternating light and dark phases, were applied to the films. The chemical changes due to the oxidative processes were quantified by determining the carbonyl index using FT-IR spectroscopy. The contact angle was used to establish variations in the hydrophobic properties of the film, while analysis of the aged films by gel-permeation chromatography allowed the acquiring of values of molecular mass of the fragments formed because of the ageing process. The work aimed to answer two key questions: (i) How do physicochemical properties of plastic change as a result of different artificial weathering protocols? (ii) Are there any specific characteristics of the material that can be used to predict degradation? The data and results obtained are presented in the next section.

## 2. Results and Discussion

Understanding how different weathering protocols influence the physicochemical properties of polymers is critical for accurately assessing their potential environmental impact. Artificial weathering protocols involving constant UV irradiation of plastics at a fixed temperature are among the most widely used methods in environmental research, employed to test the durability of plastics [33], quantify the formation of microplastics [29], and assess the eco-toxicity of weathered microplastics [14,34,35]. Given the resistance of standard plastic to oxidation and general weathering, low-density polyethylene films containing a pro-oxidant masterbatch (oxo-LDPE) were selected as a model material to accelerate abiotic degradation and evaluate the effects of weathering protocols on the physicochemical properties of the material. Evidence from prior research suggests that the abiotic degradation of LDPE and other polyolefins occurs under UV irradiation, albeit at a slow rate [17,36,37]. Chamas et al. have estimated the average surface degradation rate of six common plastics (PET, PP, HDPE, LDPE, PS, PVC) under sunlight exposure to be around 10 µm year^−1^ [38]. Similarly, long-term field studies report that while some physical fragmentation may occur, significant chemical degradation remains limited after years of environmental exposure [39]. Given these limitations and the long timescales required to observe meaningful changes, we used oxo-LDPE to expedite the weathering process and systematically analyse how different protocols impact the material’s properties.

Different weathering conditions were chosen, with the first one being the use of constant UV-A irradiation, a widely applied method described in ASTM D5208 [30], which simulates prolonged exposure to sunlight under controlled conditions. A different artificial weathering protocol was recently made available, specifically for oxo-biodegradable plastics, where plastic samples are exposed to alternating periods of UV irradiation and darkness. This is described in the PAS 9017:2020 [40] specification by the British Standards Institution. It was reported that 14 days of weathering using this protocol approximated 90 days of outdoor exposure in Florida for the polyethylene film studied [41]. Although a small number of studies have reported the use of weathering cycles [41,42], the majority of the literature data are based on weathering methods involving constant UV irradiation. There is still a significant gap in understanding the impact of the dark period in artificial weathering and how these different conditions influence the changes in the chemical structure and the physicochemical properties of the degraded plastic.

**Figure 1:**
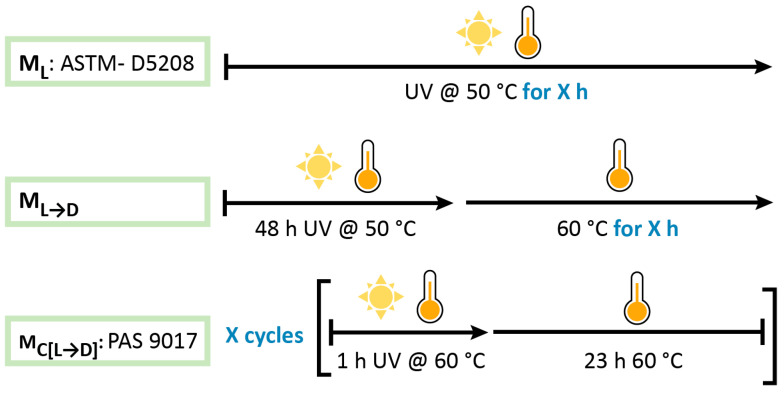
Schematic representation of weathering protocols used for artificial ageing of oxo-LDPE; X—varied time of weathering in hours for Methods A and B or number of cycles in Method C. Changes in physicochemical properties of plastic films were analysed at different time points of the weathering process.

In this study, we compared three different artificial weathering protocols, which are schematically depicted in Figure 1. Method M_L_ is based on the ASTM D5208 standard and applies continuous UV-A irradiation to plastic samples at 50 °C for an extended period. Method M_C[L→D]_ is based on the weathering cycle described in the PAS 9017:2020 and involves exposing plastic samples to cycles of light (1 h of UV-A irradiation at 60 °C) followed by a dark period (23 h at 60 °C). Method M_L→D_ represents an alternative protocol, aiming to evaluate the impact of a dark period at 60 °C applied for varying durations following an initial UV-A exposure of plastic sample for 48 h at 50 °C. This approach aligns with practices described in ASTM D5510.

### 2.1. Changes in Surface Chemistry of Oxo-LDPE Under Various Artificial Weathering Protocols

Transparent oxo-LDPE films, approximately 50 µm in thickness, were artificially aged in a UV-A weathering chamber using the three different protocols. In the first experiment, the process of abiotic degradation was monitored by analysing changes in surface chemistry using ATR-FTIR spectroscopy. This technique provides a simple, convenient, and rapid method for characterising surface changes resulting from artificial weathering. It is widely employed in environmental research and the analysis of weathered plastics, to cite a few [11,43,44]. The weathering processes proceed via an oxidative process that results in changes in the functional groups, with the introduction of alcohols, aldehydes, ketones and carboxylic acids, to name a few (see Figure S1 for mechanisms).

**Figure 2:**
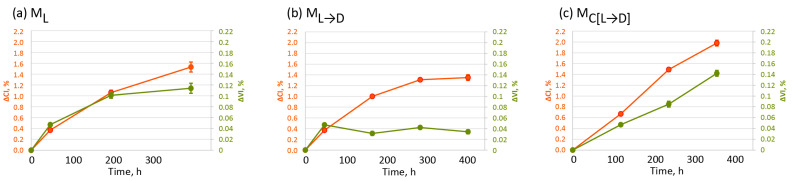
Changes in surface chemistry of oxo-LDPE due to weathering with Methods A, B, C (**a**–**c**), measured at different time points; ∆CI, % (orange, left y-axis)—changes in carbonyl index compared to unaged LDPE; ∆VI, % (green, right y-axis)—changes in vinyl index compared to unaged LDPE. Error bars represent standard errors for eight repeats.

The oxidation of oxo-LDPE can be quantified by analysing the growing broad peak in the carbonyl region in the FTIR spectra (see Figure S2 for the spectrum of unaged and artificially aged—15 days—oxo-LDPE films with method M_C[L→D]_). This peak encompasses overlapping signals from various functional groups, including ketones at 1718 cm^−1^, carboxylic acids at 1715 cm^−1^, aldehydes at 1734 cm^−1^, esters at 1740 cm^−1^, and lactones at 1780 cm^−1^. The carbonyl index is a key parameter used to assess overall surface oxidation. For this study, the carbonyl index of artificially weathered oxo-LDPE films was determined using the specified area under the band methodology established by Almond et al. [45] and described in PAS 9017:2020 [8]. It is calculated as the ratio between the area under the carbonyl band (1850–1650 cm^−1^) and the C-H band (1500–1420 cm^−1^). The vinyl index was determined as the intensity of the vinyl band at 908 cm^−1^ divided by the maximum peak height of a C-H reference peak in the 1480–1450 cm^−1^ region, corresponding to aliphatic C-H stretching common to all oxo-LDPE samples. Similar methods have been employed in studies by Guadagno et al. [46] and Yagoubi et al. [47]. Figure 2 summarises the changes in carbonyl index (ΔCI) and vinyl index (ΔVI) obtained when comparing aged to unaged oxo-LDPE, resulting from the artificial weathering of plastic samples with the three different protocols. The individual FTIR spectra for unaged and aged samples under all the different protocols are available in Figure S3.

Oxidation of the surface of oxo-LDPE films occurred under all three weathering protocols investigated. The highest surface oxidation was observed with the M_C[L→D]_ protocol, reaching a ΔCI value of 1.98 ± 0.05 after 360 h of exposure. This is particularly noteworthy given that this method involves the shortest total UV irradiation time (5–15 h) compared to 48–400 h in M_L_ and 48 h in M_L→D_. This increased oxidation can be partially attributed to the 10 °C higher temperature during UV irradiation in M_C[L→D]_ compared to M_L_ (60 °C and 50 °C, respectively), as the Arrhenius law predicts that a 10 °C increase approximately doubles the rate of chemical reactions [48]. However, considering the substantial 26-fold difference in UV exposure time between M_C[L→D]_ and M_L_, the temperature increase alone does not fully account for the higher surface oxidation achieved with M_C[L→D]_ (ΔCI of 1.98 ± 0.05 after 360 h) compared to M_L_ (ΔCI of 1.53 ± 0.09 after 400 h). This finding suggests that while UV exposure is crucial to initiate oxidation and generate radicals, the propagation of the reaction and continued oxidation can progress during the dark period. Supporting this, data from the M_L→D_ protocol indicate that oxidation continues during the dark phase following 48 h of UV exposure, as evidenced by an increase in ΔCI from 0.37 ± 0.02 to 1.35 ± 0.05 after 408 h of artificial weathering. However, the oxidation rate slows and eventually reaches a plateau observed after 288 h. In contrast, the cyclic alternation of light and dark phases in the M_C[L→D]_ method results in a linear increase in oxidation levels, highlighting the unique efficacy of this protocol in achieving significant levels of plastic weathering.

The number of vinyl groups, represented by the Vinyl Index (ΔVI), increased under all three weathering protocols, but the extent of the increase varied significantly depending on the method. The M_C[L→D]_ protocol resulted in the highest number of vinyl groups, with a ΔVI of 0.142 ± 0.005 after 360 h of exposure. In comparison, the M_L_ protocol showed minimal change in ΔVI between 200 and 400 h of UV exposure, with values of 0.101 ± 0.005 and 0.114 ± 0.009, respectively. For the M_L→D_ protocol, a distinct trend was observed. After an initial rise in ΔVI to 0.047 ± 0.002 following 48 h of UV exposure, the values stabilised during the sequential dark period at 60 °C, fluctuating between 0.031 ± 0.004 and 0.042 ± 0.002. This pattern indicates that while surface oxidation continues without UV irradiation, the formation of vinyl groups is closely linked to higher carbonyl indexes and dependent on the presence of regular cyclic UV exposure as a result of the type II Norrish reaction occurring on terminal methyl ketones (see Figure S1).

In summary, artificial weathering under all three protocols resulted in measurable oxidation and the formation of vinyl groups, with the M_C[L→D]_ protocol showing the most pronounced changes. These results provide evidence of the importance of periodic UV exposure in driving surface chemical transformations. To gain a more comprehensive understanding of the impact of different weathering protocols, the next section will report on changes in the physicochemical properties of the aged plastic and discuss any correlation between said properties and the surface chemistry of the fragments.

### 2.2. Physicochemical Changes in Oxo-LDPE with Different Weathering Protocols

**Figure 3:**
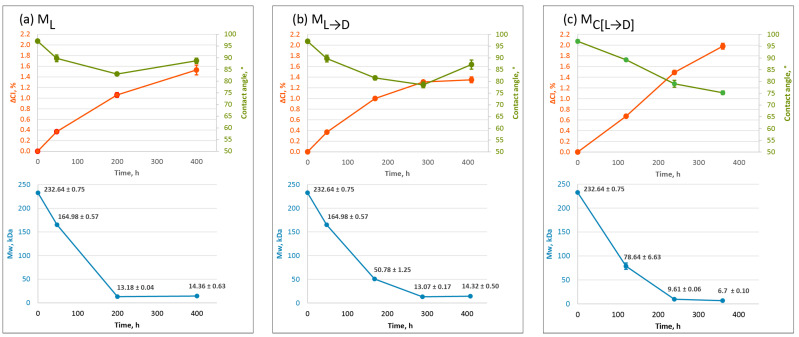
Changes in physicochemical properties of oxo-LDPE due to weathering with methods M_L_, M_L→D_, M_C[L→D]_ (**a**,**b**,**c**, respectively), measured at different time points; top graph, orange, left y-axis—changes in carbonyl index compared to unaged oxo-LDPE (∆CI, %) (*n* = 8)—error bars indicated standard error; top graph, green, right y-axis—water contact angle (Contact angle, °) (*n* = 8) —error bars indicated standard error; bottom graph, blue—weight average molecular weight (Mw, kDa), measured by GPC (*n* = 2)—error bars indicate standard deviation.

Surface wettability and changes in polymer molecular weight are parameters commonly used to characterise plastic degradation [17,18,24,25]. Surface wettability, measured by analysing the contact angle of water droplets on plastic films, is directly related to surface hydrophobicity. Typically, it decreases as surface oxidation occurs during UV irradiation, indicating a shift toward greater surface polarity [24]. Meanwhile, the molecular weight of plastics significantly decreases during weathering, primarily as a result of chain scission driven by photochemical degradation and Norrish reaction [49,50]. Weight-average molecular weights (Mw) were obtained via gel permeation chromatography (GPC) equipped with a triple-detection system (refractive index, light scattering, and viscometer detectors) and calibrated with polystyrene standard. GPC allowed the evaluation of the differences in Mw between weathered oxo-LDPE samples. To ensure the reproducibility of the Mw data, the characterisation was repeated twice. Examples of molecular weight distributions are presented in Figure S4, while mean molecular weights, as well as polydispersity indices, are summarized in Table S1. It is important to note that GPC analysis provides distribution curves and values of polydispersity to allow comparison between the materials. Figure 3 presents data on surface oxidation (ΔCI), surface wettability (water contact angle), and Mw for oxo-LDPE samples weathered under different protocols. Comparing these parameters simultaneously provides valuable insights into how physicochemical changes in the material correlate under different weathering conditions. The measurements of the contact angle for unaged and aged oxo-LDPE samples under varied artificial weathering conditions (Table S1), images of the water drop used for contact angle calculations (Figure S4), examples of molecular weight distributions (Figure S5) and mean molecular weight distributions of all samples used in the paper (Table S2) are available in the Supplementary Materials.

Under method M_L_, continuous UV irradiation resulted in a general increase in surface oxidation and hydrophilicity, alongside a substantial reduction in molecular weight. However, after 200 h of exposure, notable changes in the trends of surface wettability and molecular weight were observed. Under UV exposure, the water contact angle initially decreased from 97.08 ± 0.66° to 83.03 ± 0.51° after 200 h and then increased to 88.69 ± 1.11° after 400 h. The slight increase in contact angle suggests some minor changes in the surface chemistry that impact the hydrophobic character of the materials. Interestingly, when the molecular weight was analysed, a somewhat similar trend was observed. The molecular weight decreased sharply from 232.64 ± 0.75 kDa in the unaged sample to 13.18 ± 0.04 kDa after 200 h of UV exposure, representing a 94% reduction. However, between 200 and 400 h, the molecular weight exhibited comparatively small changes, although showing a slight increase to the value of 14.36 ± 0.63 kDa. This may result from crosslinking reactions occurring alongside chain scission during prolonged UV exposure, leading to a slightly increased molecular weight and higher surface hydrophobicity [49].

A similar pattern was observed for method M_L→D_, where surface hydrophilicity and molecular weight decreased during the first 300 h of weathering, followed by increases in both. Thus, the water contact angle decreased to 78.53 ± 1.23° at 288 h but then rose to 87.17 ± 1.96° after 400 h. Similarly, the molecular weight dropped to 13.07 ± 0.17 kDa at 288 h and subsequently showed a very small increase to 14.32 ± 0.5 kDa after 408 h. Despite the substantially shorter UV exposure in M_L→D_ (48 h versus 400 h in M_L_), the degradation rates based on changes in molecular weight of oxo-LDPE were similar, further supporting the notion that radicals generated during the initial UV irradiation phase promote continued degradation in the dark period.

For oxo-LDPE samples, weathered using method M_C[L→D]_, the observed trends differed notably. Surface oxidation exhibited a continuous linear increase, while surface hydrophilicity steadily decreased, reaching a minimum water contact angle of 75.21 ± 0.74° after 360 h of exposure. Molecular weight also declined to the lowest recorded value of 6.7 kDa, falling below the 10 kDa threshold commonly considered indicative of low-molecular-weight polymers or oligomers [51,52]. These results suggest that periodic 1-h UV irradiation generates sufficient radicals to sustain degradation during the dark cycle, with chain scission reactions predominating over crosslinking throughout the studied exposure period.

**Figure 4:**
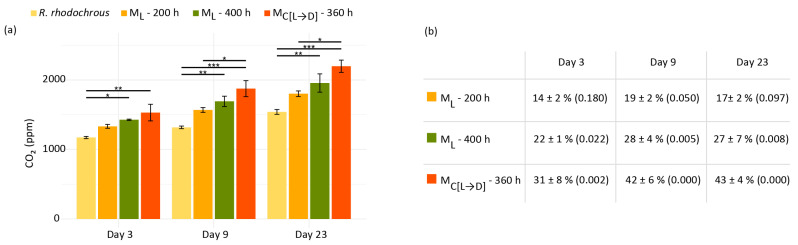
Biodegradation of weathered oxo-LDPE samples was measured as CO_2_ production from *R. rhodochrous* (*n* = 3); (**a**) absolute values of CO_2_ production (ppm) measured with GC after incubation of bacteria alone and with plastic samples for 3, 9, and 23 days. Differences among samples at each time point were assessed by one-way ANOVA followed by Tukey’s multiple comparisons test; significance is indicated by * (*p* < 0.05), ** (*p* < 0.01) and *** (*p* < 0.001). (**b**) increase in CO_2_ production (average ± SE, %) when *R. rhodochrous* was grown on plastics compared to alone; oxo-LDPE was aged under constant UV irradiation for 200 h (M_L_—200 h), 400 h (M_L_—400 h) and under weathering cycles (M_C[L→D]_—360 h). Adjusted *p*-values from Tukey’s post-hoc test are shown in brackets.

These findings highlight the impact of weathering protocols on the interplay of surface and molecular changes in oxo-LDPE, demonstrating the complex dynamics of photodegradation and oxidation processes. To further explore the implications of these physicochemical changes, we investigated the bacterial biodegradation of selected oxo-LDPE samples using the method established by Rose et al. [26]. Figure 4 presents the concentration of CO_2_ produced, as measured by GC-MS when *R. rhodochrous* was incubated alone and with oxo-LDPE samples weathered using methods M_L_ and M_C[L→D]_. Three samples exhibited high levels of surface oxidation (ΔCI > 1), reaching a threshold value considered indicative of biodegradability according to PAS 9017.

The presence of weathered samples in the cultures resulted in increased CO_2_ production by *R. rhodochrous*, which is indicative of their biodegradation taking place, given that the assay is carried out in such a way that plastic samples are the only source of carbon (Table S3). Notably, an 8–10% increase in CO_2_ production was observed after 23 days for bacterial growth on M_L_—200 h compared to M_L_—400 h, correlating with higher surface oxidation (ΔCI of 1.06 ± 0.04 vs. 1.53 ± 0.09, respectively). Sample M_C[L→D]_—360 h showed the highest proportion of biotic degradation, with a 31–43% increase in CO_2_ release compared to the *R. rhodochrous* control and a 9–16% increase compared to sample M_L_—400 h. This enhanced degradation is likely attributable to a combination of lower molecular weight, higher surface hydrophilicity, and increased surface oxidation.

Overall, the results emphasise the multifaceted nature of plastic degradation, where surface oxidation, molecular weight reduction, and hydrophilicity contribute collectively to biodegradation outcomes. While the interplay of these properties provides valuable insights, it also raises a question on the potential limitations of relying on a single metric to comprehensively assess degradation.

### 2.3. Can Plastic Degradation Be Accurately Assessed Through a Single Metric?

To attempt to answer the question, three pairs of aged samples were selected for further evaluation, with each pair sharing one similar value for one of the parameters (contact angle, carbonyl index, weight average molecular weight) and a different one for the second one, despite being obtained via different protocols.

**Figure 5:**
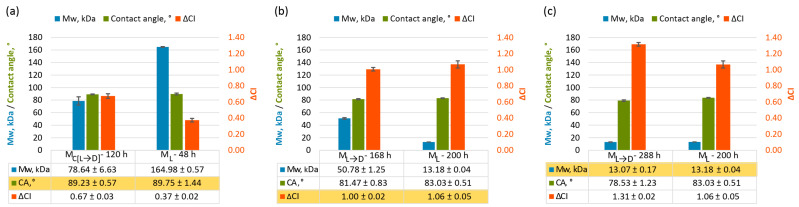
Comparison of physicochemical characteristics (contact angle, ° (CA); changes in carbonyl index compared to unaged oxo-LDPE (∆CI); weight average molecular weight (Mw, kDa)) of oxo-LDPE samples weathered at different conditions: (**a**) 120 h or weathering with method M_C[L→D]_ and 48 h with M_L_, (**b**) 168 h or weathering with method M_L→D_ and 200 h with M_L_, (**c**) 288 h or weathering with method M_L→D_ and 200 h with M_L_. The X-axis reports the weathering method M_L_, M_L→D_, M_C[L→D]_ and the time point of the analysis (48–200 h), and the Y-axis reports the Mw (blue), contact angle (green) and ∆CI (orange). Error bars indicate standard error (*n* = 8) for ∆CI and contact angle and standard deviation for Mw (*n* = 2).

In Figure 5a, two oxo-LDPE samples weathered under different conditions display similar surface hydrophilicity, as indicated by comparable water contact angles ~89°. However, their molecular weights present notable differences (78.64 ± 6.63 kDa vs. 164.98 ± 0.57 kDa), along with their degree of surface oxidation (ΔCI of 0.67 ± 0.03 vs. 0.37 ± 0.02). This demonstrates that even when surface wettability appears unchanged, underlying molecular changes can vary markedly, reflecting distinct stages of degradation. This can be the result of fragmentation of the plastic, with loss of microplastics and volatile compounds, leaving the material with similar functional groups on the surface. A different trend emerges in Figure 5b, where two samples with comparable surface oxidation levels (ΔCI ~1) exhibit significantly different molecular weights (50.78 ± 1.25 kDa vs. 13.18 ± 0.04 kDa). Although their hydrophilicity, measured by water contact angle, is only slightly different (81.47 ± 0.83° vs. 83.03 ± 0.51°), the disparity in molecular weight indicates that surface oxidation alone cannot fully account for the degree of degradation. Figure 5c further highlights these complexities by comparing two samples with similar molecular weights (~13 kDa). Despite this shared characteristic, their surface hydrophilicity and oxidation levels differ considerably. The water contact angle is 83.03 ± 0.51° for the sample weathered by the M_L_ method (200 h), compared to 78.47 ± 1.24° for the sample weathered by the M_L→D_ (288 h). Correspondingly, surface oxidation (ΔCI) is higher for the M_L→D_ sample (1.31 ± 0.02) than for the M_L_ sample (1.06 ± 0.04). As the biodegradation experiments suggest, higher surface oxidation correlates with enhanced bacterial degradation, underscoring the critical role of surface chemistry in plastic degradation.

These findings collectively provide evidence that the degradation of plastic is a complex phenomenon influenced by multiple factors and that no single metric—whether surface hydrophilicity, molecular weight, or surface oxidation—can fully describe the degradation process. Instead, an integrated, multi-parametric approach is necessary to understand the complex interplay of these properties and their combined effects on the environmental behaviour of plastics and their biodegradability.

## 3. Materials and Methods

### 3.1. Materials

Oxo-low-density polyethylene (oxo-LDPE) films of ~50 µm thickness were supplied by Symphony Environmental Technologies (Borehamwood, UK). They were manufactured from unstabilised Total 12022 FN 24 LDPE resin with manganese stearate as a catalyst, iron (III) stearate as a photo and Irganox 1010 as a phenolic antioxidant. Bacterial strain *Rhodococcus rhodochrous* (ATCC-29672) was supplied by LGC Ltd. (Teddington, UK). Chemicals used in the preparation of the liquid media for *R. rhodochrous* (ammonium iron (II) sulfate hexahydrate, calcium chloride hexahydrate, zinc sulfate heptahydrate and cobalt (II) nitrate hexahydrate) were supplied by Fisher Scientific UK Limited (Dartford, UK) while manganese (II) sulfate tetrahydrate, sodium molybdate dihydrate were purchased from Merck Life Science UK Limited (Glasgow, UK).

### 3.2. Accelerated Artificial Weathering

Accelerated weathering of oxo-LDPE films was conducted using a Q-LAB QUV accelerated weathering tester equipped with UVA-340 lamps (Q-Lab Europe Ltd., Bolton, UK) with an emission peak at 340 nm and irradiance of 0.78 W·m^−2^·nm^−1^. The controlled black-panel temperature was set at 50 °C or 60 °C. In method M_L→D_, thermal ageing was conducted in a GENLAB Classic Oven (Genlab Ltd., Cheshire, UK) at 60 °C or in a Memmert UFE 600 fan-assisted oven at 60 °C in accordance with ASTM D5510 Procedure B: Forced Ventilation Oven. The weathering of oxo-LDPE films was carried out with the samples flattened and fixed on a metal holder in the absence of humidity.

### 3.3. Analysis of Surface Chemistry

Changes in the surface chemistry of plastic films were investigated using ATR-FTIR spectroscopy. Spectra were recorded on a Thermo-scientific Nicolet IS10 (Thermo Fisher Scientific (PN1) UK Ltd., Waltham, MA, USA) or Shimadzu ATR-FTIR (Shimadzu Corp., Kyoto, Japan) spectrometers equipped with diamond crystals. OMNIC Paradigm v2.3, LabSolution v2.25 and TQ Analyst EZ Edition v9.14.327 software were used for the analysis. The spectra scans were recorded at 16 scans summation and 4 cm^−1^ resolution in a range of 4000–650 cm^−1^. The CI was calculated using the specified area under band method reported by Almond et al. [45] and codified in BSI PAS 9017, where the CI value is the ratio of the area band at 1850–1650 cm^−1^ divided by the area under band at 1500–1420 cm^−1^. VI was calculated as the maximum peak height at 907–910 cm^−1^ divided by the maximum C-H peak height in the area 1470–1450 cm^−1^. The data for ∆CI and ∆VI were calculated by subtracting the CI and VI values of the unaged plastic samples. Each value represents the mean of eight measurements, presented with the corresponding standard error (±SE).

### 3.4. Analysis of Surface Hydrophilicity

Changes in hydrophobicity or wettability of sample surfaces were measured using a semi-automatic wettability analysis DSA25S Drop Shape Analyzer KRÜSS system with an attached Basler camera (Krüss Optronic GmbH, Hamburg, Germany). The wetting liquid was set with 2.0 μL droplets of distilled water from the PURELAB Water Purification System (Purelab Aesthetics Ltd., Wembley, UK) at a rate of 2.67 μL·s^−1^ at room temperature. The contact angle values were determined using images of each droplet obtained with KRÜSS Scientific ADVANCE Drop Shape software (v1.13.0.21301) for at least ten measurements for each sample with an ellipse fitting method and automatic baseline.

### 3.5. Analysis of Molecular Weight

The molecular weight of the plastic samples was determined using an Agilent PL-220 high-temperature GPC system (Agilent Technologies Inc., Santa Clara, CA, USA). The system was equipped with differential refractive index, light scattering, and viscometer detectors. It utilized two Olexis PL-Gel columns (13 µm, 300 × 7.5 mm) and an Olexis PL-Gel guard column (13 µm, 7.5 × 50 mm), calibrated with polystyrene standards (Agilent Easivials, Agilent Technologies Inc., Santa Clara, CA, USA) with molecular weights ranging from 580 to 6,570,000 g·mol^−1^.

Measurements were conducted using 1,2-dichlorobenzene as a solvent, with 250 ppm butylated hydroxytoluene as the eluent, at 140 °C and a flow rate of 1 mL·min^−1^. Prior to injections, the samples were prepared on a known concentration and were heated at 150 °C for 3 h before filtering using a hot filtration device, PL-SP 260 VS (Agilent Technologies Inc., Santa Clara, CA, USA). Samples were run in duplicates and data such as weight average molecular weight (Mw), peak molecular weight (Mp), number average molecular weight (Mn), polydispersity indices were reported as mean of the duplicates ± corresponding standard deviation.

### 3.6. Analysis of Bacterial Biodegradation

*Rhodococcus rhodochrous* were cultured in media as recommended, specifically using tryptone soya broth (Oxoid, Hampshire, UK) [26]. Plastic films were hole-punched to a size of 3 mm in diameter, sterilized with 70% ethanol, and allowed to dry prior to use. All bacterial handling was conducted in a class II biosafety cabinet, with laboratory equipment disinfected using Virkon solution before each use.

*R. rhodochrous* was grown at 30 °C under shaking conditions at 120 rpm. Prior to incubation with plastic, the bacteria were cultured overnight in minimal media supplemented with 1% glucose solution. The cells were pelleted by centrifugation at 4000 rpm for 5 min and washed in fresh minimal media to remove residual carbon, and this process was repeated at least three times. The resulting bacterial solution was diluted in minimal media to achieve an optical density at 600 nm of 0.2, as described by Rose et al. [26].

A 0.4 mL volume of the prepared media was added to each autoclaved exetainer containing the ethanol-washed plastic samples. Negative controls included exetainers containing only media or a media/bacteria mix without additional carbon sources. Positive controls were prepared by adding 20 µL of a 20% glucose stock solution to the minimal media alone or to the media/bacteria mix. Five replicates of each experimental condition were prepared in 12 mL gas-tight vials with screw caps and pierceable chlorobutyl rubber septa (Labco exetainers, Ceredigion, UK). The sealed vials were incubated at 30 °C with shaking at 120 rpm to maintain continuous movement of the cultures.

The release of CO_2_ during bacterial growth was measured using gas chromatography (GC) analysis. A syringe connected to an autosampler (Multipurpose Sampler MPS2, Gerstel GmbH, Mülheim an der Ruhr, Germany) extracted a 100 μL sample from the vial’s headspace during analysis. This sample was injected into a gas chromatograph equipped with a flame ionization detector (GC-FID) and a hot nickel catalyst (Agilent Technologies UK Limited, Cheshire, UK). The GC system was configured with the column temperature set at 30 °C, the detector at 375 °C, and the catalyst at 385 °C. CO_2_ separation was achieved using a stainless-steel column (6’ × 1/2ø) packed with Porapak Q (80/100 mesh) and a hydrogen/air mixture (7% hydrogen, 93% zero-grade air, BOC, London, UK) as the carrier gas at a flow rate of 430 mL/min.

The headspace concentrations of CO_2_ and CH_4_ were determined from peak areas using an electronic integrator. These values were initially displayed as CO_2_/area and then converted to ppm using a linear regression method in Excel. Final CO_2_ concentrations were calibrated against fresh standards of air and calibration gas containing 100 ppm CH_4_ and 3700 ppm CO_2_, balanced with nitrogen, prepared on the same day as the analysis. Statistical analysis of data on CO_2_ release was performed in R (version 4.4.2; R Core Team, Vienna, Austria) using the dplyr, ggplot2, and rstatix packages. Group comparisons were assessed using one-way ANOVA followed by Tukey’s post-hoc test, with a significance threshold of *p* < 0.05.

## 4. Conclusions

The findings of this study provide evidence of the critical role of artificial weathering protocols in influencing the degradation pathways and physicochemical properties of oxo-LDPE. Through a systematic comparison of samples aged via continuous UV-A irradiation (M_L_), cyclic UV-dark exposure (M_C[L→D]_), or sequential UV-dark phases (M_L→D_), it was evident that each protocol uniquely influenced oxidation, surface properties, and molecular weight. Notably, the M_C[L→D]_ protocol, with its alternating light and dark phases, elicited the highest degree of surface oxidation despite reduced overall UV exposure time, highlighting the continued oxidative reactions occurring during dark phases. This result points to the significant role of environmental conditions in plastic degradation and microplastic formation.

Surface properties, including hydrophilicity and vinyl group formation, were intricately linked to weathering conditions. Increased carbonyl content enhanced surface hydrophilicity, which may affect interactions with biological and environmental systems. Vinyl group formation required sustained or cyclic UV exposure, reflecting the complexity of photochemical reactions under varying conditions. Biodegradation experiments demonstrated that weathered oxo-LDPE samples with higher surface oxidation levels (ΔCI > 1) supported increased CO_2_ production by *Rhodococcus rhodochrous*, with the M_C[L→D]_—360 h protocol yielding the highest biodegradation rates—31–43% higher than the control. This enhanced degradation is likely driven by a synergistic effect of reduced molecular weight, elevated surface hydrophilicity, and increased oxidation. These findings enhance our understanding of how environmental factors influence plastic weathering and biodegradability, providing a foundation for more accurate simulation of real-world scenarios.

Overall, this research highlights the importance of tailoring experimental weathering protocols to specific applications, particularly for environmental risk assessments and material design. The cyclic weathering emerged as a closer approximation to natural conditions, offering valuable insights into the degradation processes occurring in outdoor environments. The findings of this study have broader implications, including improving the standardisation of biodegradability assessments, enhancing our understanding of environmental impacts, and informing policies for managing plastic waste and developing more sustainable materials.

## Data Availability

The raw data supporting the conclusions of this article will be made available by the authors upon request.

## Supplementary Materials

The following supporting information can be downloaded at: https://www.mdpi.com/article/10.3390/polym17131798/s1. Figure S1: Mechanism of photooxidation of aliphatic polymers under aerobic conditions; Figure S2: ATR-FTIR spectra for oxo-LDPE unaged and aged under 400 h of UV irradiation at 50 °C; Figure S3: ATR-FTIR spectra of oxo-LDPE unaged and aged under UV irradiation 50 °C for 48 h (UV48), 200 h (UV200), and 400 h (UV400), under UV irradiation for 48 h followed by heat only 60 °C for 120 h (UV48t120), 240 h (UV48t240) and 360 h (UV48t360), and with cycle 60 °C for 5 days (C5—120 h), 10 days (C10—240 h) and 15 days (C15—360 h). Average for eight replicates; Table S1: Mean molecular weight distribution (in kDa) of all unaged and artificially aged samples used in the work presented in Section 3, obtained by conventional analysis from HT-GPC using 1,2-dichlorobenzene solvent. Average of two replicates ± standard deviation (sd); Figure S4: Molecular weight distribution (MWD) for oxoLDPE unaged (a), aged under 48-h-photooxidation followed by heat for 360 h (b) and with 15 days of cycle (c) for one replicate. Data set and graph are provided for clarity; Figure S5: Contact angle measurements (°) for oxoLDPE unaged (a) and aged under UV irradiation 50 °C for 48 h (b), 200 h (c), and 400 h (d), under UV irradiation for 48 h followed by heat only 60 °C for 120 h (e), 240 h (f) and 360 h (g), and with cycle 60 °C for 5 days (h), 10 days (i) and 15 days (j); Table S2: Contact angle (CA) measured for unaged and aged oxo-LDPE samples weathered under varied conditions, the data represent an average value with standard error (*n* = 8); Table S3: CO_2_ production (ppm) from *Rhodococcus rhodochrous* grown alone and on oxo-LDPE samples aged under UV irradiation at 50 °C for 200 h (UV_200_) and 400 h (UV_400_), and with cycle 60 °C for 15 days (C_15_-360 h). Average of three replicates ± standard error.
