# Supplementary material for: The Role of Artificial Weathering Protocols on Abiotic and Bacterial Degradation of Polyethylene
Source: Polymers (Basel). 2025 Jun 27;17(13):1798. doi: 10.3390/polym17131798 (PMC12251912; doi:10.3390/polym17131798)
Supplement: Supplementary file 1 [file polymers-17-01798-s001.zip › polymers-3667774-supplementary.pdf]

Supplementary material

The role of Artificial Weathering Protocols on Abiotic and Bacterial Degradation of Polyethylene

Pauline F. De Bigault De Cazanove [1], Alena Vdovchenko [1], Ruth S. Rose [2] and Marina Resmini [1,*]

[1] Department of Chemistry, School of Physical and Chemical Sciences, Queen Mary University of London, Mile End road, London E1, 4NS;

[2] School of Biological and Behavioural Sciences, Queen Mary University of London, Mile end road, London E1 4NS;

* Correspondence: m.resmini@qmul.ac.uk

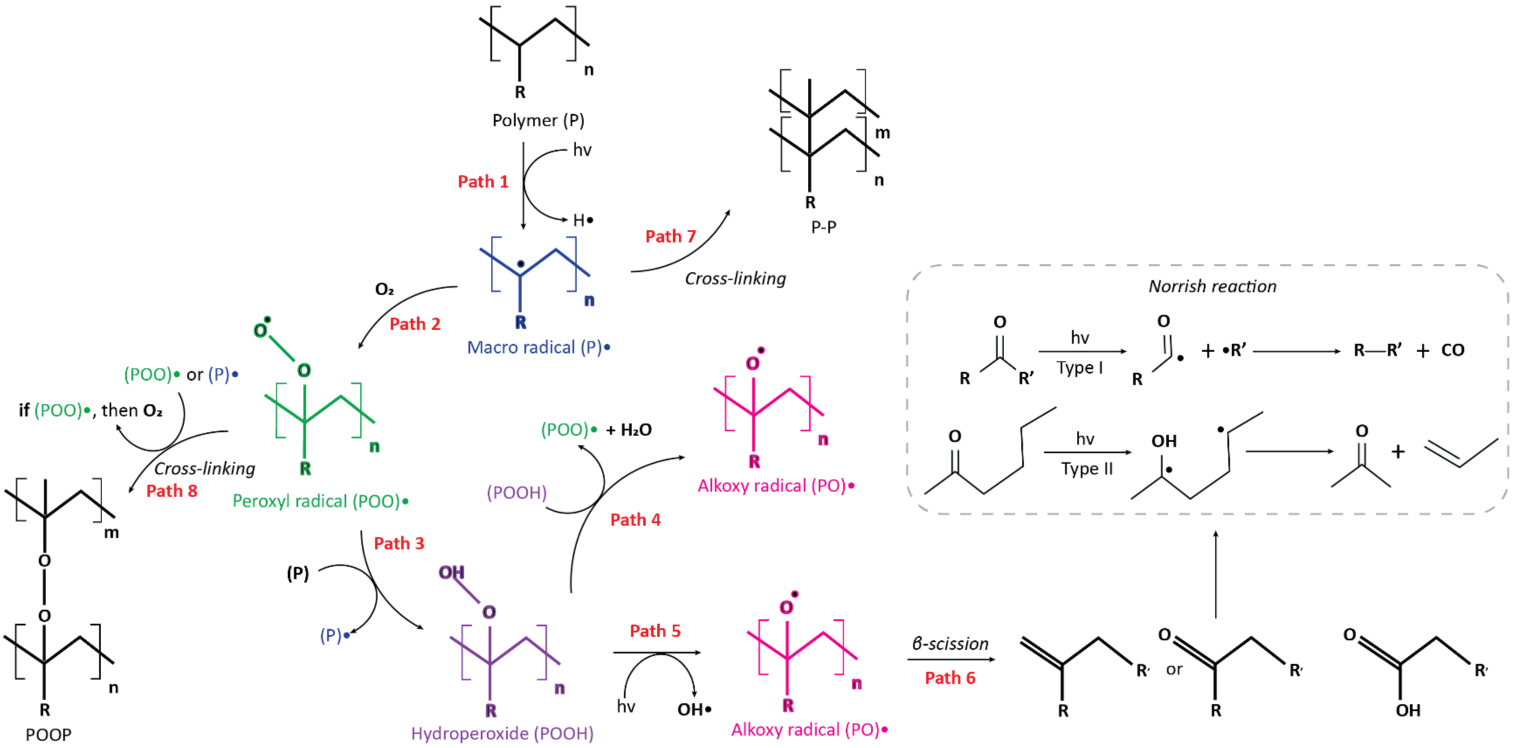

Figure S1. Mechanism of photooxidation of aliphatic polymers under aerobic conditions

Specified Area Under Band Method

The Specified Area Under band method (SAUB) established by *Almond, et al.* is calculated as the ratio between the area under the band of the carbonyl peak area (1850-1650 cm^{-1}) and the C-H peak area (1500-1420 cm^{-1}) (Equation 1).

$$\text{CI SAUB} = \frac{C{=}O\ Area\ Under\ Band\ (1850{-}1650\ cm^{-1})}{C{-}H\ Area\ Under\ Band\ (1500{-}1420\ cm^{-1})} \quad \textbf{(Equation 1)}$$

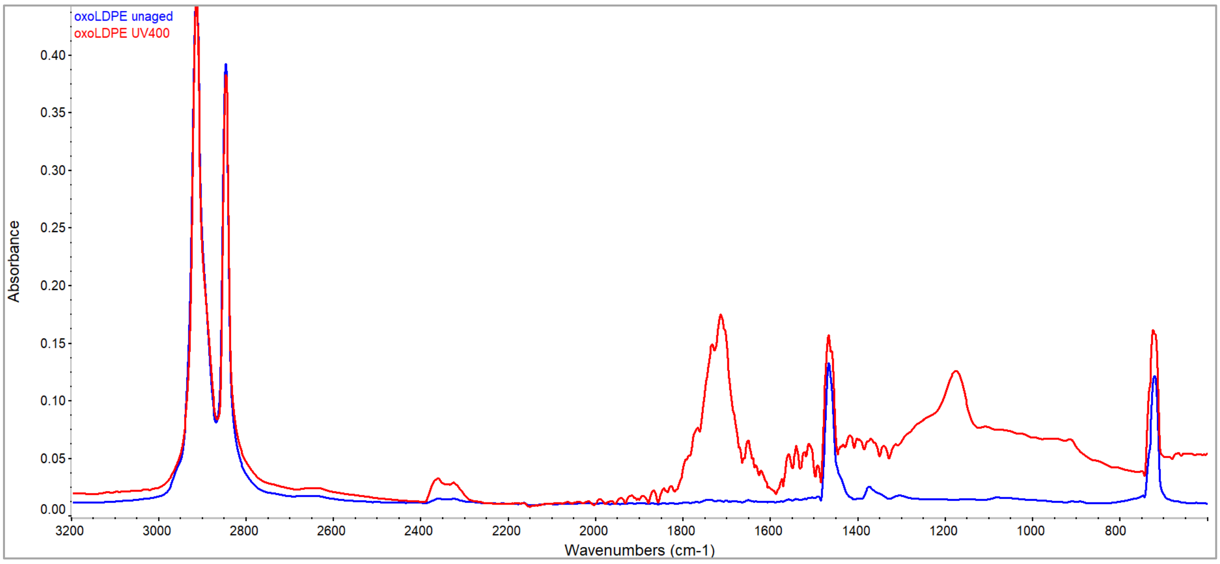

Figure S2. ATR-FTIR spectra for oxoLDPE unaged and aged under 400 h of UV irradiation at 50 °C.

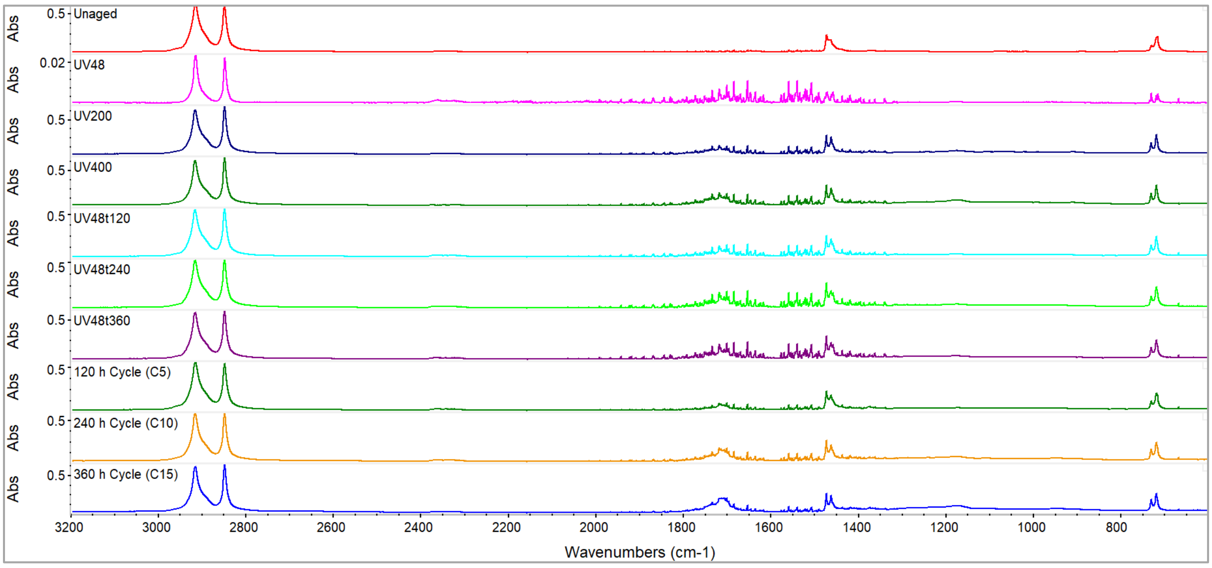

Figure S3. ATR-FTIR spectra for oxoLDPE unaged and aged under UV irradiation 50 °C for 48 h (UV48), 200 h (UV200), and 400 h (UV400), under UV irradiation for 48 h followed by heat only 60 °C for 120 h (UV48t120), 240 h (UV48t240) and 360 h (UV48t360), and with cycle 60 °C for 5 days (C5 - 120 h), 10 days (C10 - 240 h) and 15 days (C15 - 360 h). Average for eight replicates.

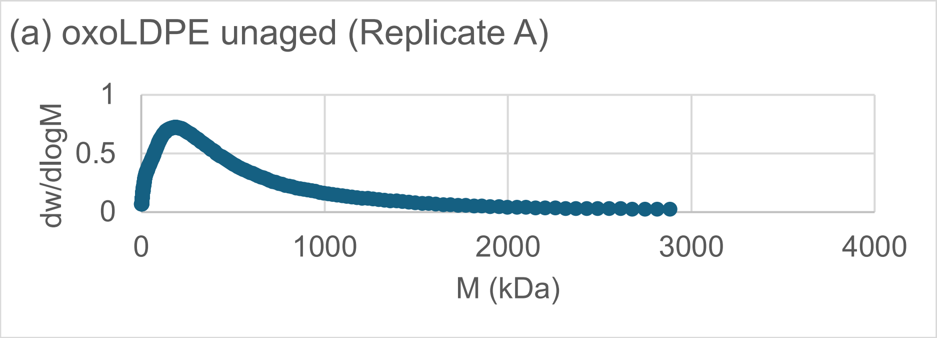

MWD	Mp	Mn	Mw	Mz	Mz+1	Mv	PD
g/mol	193206	35339	232120	681759	1252157	607581	6.568381
kDa	193.206	35.339	232.12	681.759	1252.157	607.581	6.568381

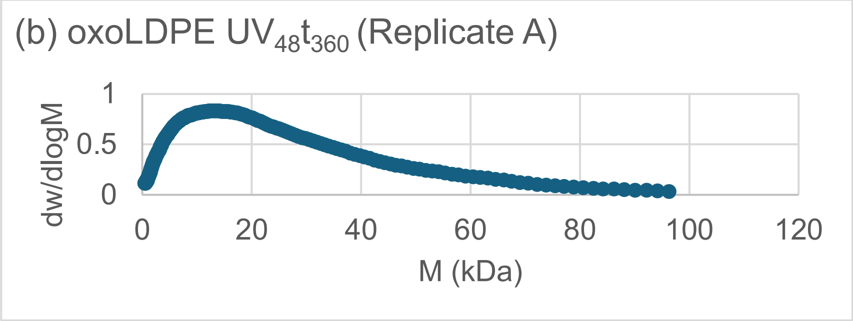

MWD	Mp	Mn	Mw	Mz	Mz+1	Mv	PD
g/mol	12776	4787	14675	29575	44013	27457	3.065594
kDa	12.776	4.787	14.675	29.575	44.013	27.457	3.065594

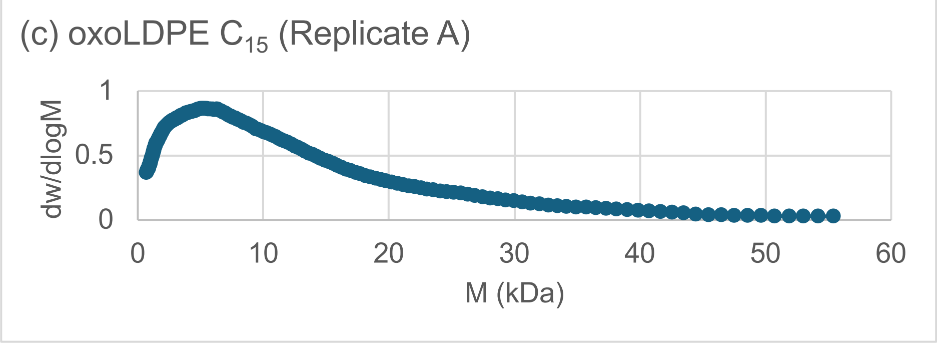

MWD	Mp	Mn	Mw	Mz	Mz+1	Mv	PD
g/mol	5339	2861	6767	14312	23127	13099	2.365257
kDa	5.339	2.861	6.767	14.312	23.127	13.099	2.365257

Figure S4. Molecular weight distribution (MWD) for oxoLDPE unaged (a), aged under 48-h-photooxidation followed by heat for 360 h (b) and with 15 days of cycle (c) for one replicate. Data set and graph are provided for clarity.

Table S1. Mean molecular weight distribution (in kDa) of all unaged and artificially aged samples used in the work presented in Chapter 3, obtained by conventional analysis from HT-GPC using 1,2-dichlorobenzene solvent. Average of two replicates ± standard deviation (sd).

Samples	Ageing conditions	Mp (kDa) ± sd	Mn (kDa) ± sd	Mw (kDa) ± sd	Mz (kDa) ± sd	Mz+1 (kDa) ± sd	Mv (kDa) ± sd	PD ± sd
oxoLDPE	Unaged	195.415 ± 3.123	36.475 ± 1.607	232.651 ± 0.750	675.169 ± 9.320	1279.969 ± 39.332	600.013 ± 10.703	6.384 ± 0.261
oxoLDPE UV_{48}	48 h UV 50 °C	145.803 ± 2.315	29.317 ± 1.089	164.978 ± 0.567	454.780 ± 2.157	803.781 ± 22.329	408.977 ± 3.485	5.631 ± 0.190
oxoLDPE UV_{200}	200 h UV 50 °C	10.731 ± 0.000	3.725 ± 0.178	13.178 ± 0.041	30.597 ± 1.186	49.997 ± 2.358	27.914 ± 1.007	3.542 ± 0.180
oxoLDPE UV_{400}	400 h UV 50 °C	10.387 ± 0.160	3.894 ± 0.236	14.356 ± 0.632	36.214 ± 4.438	64.889 ± 14.460	32.553 ± 3.452	3.689 ± 0.061
oxoLDPE $UV_{48}t_{120}$	48 h UV 50C then 120 h heat 60 °C	23.297 ± 1.080	8.921 ± 0.144	50.782 ± 1.245	198.223 ± 12.944	386.725 ± 29.194	172.261 ± 10.409	5.692 ± 0.048
oxoLDPE $UV_{48}t_{240}$	48 h UV 50C then 240 h heat 60 °C	11.709 ± 0.000	4.644 ± 0.265	13.070 ± 0.171	25.742 ± 0.538	38.419 ± 2.321	23.922 ± 0.374	2.818 ± 0.124
oxoLDPE $UV_{48}t3_{60}$	48 h UV 50C then 360 h heat 60 °C	12.917 ± 0.199	4.672 ± 0.163	14.323 ± 0.498	28.384 ± 1.685	41.689 ± 3.287	26.416 ± 1.472	3.066 ± 0.000
oxoLDPE C_5	5 days - 120 h Cycle 60 °C	23.914 ± 3.316	11.263 ± 0.459	78.639 ± 6.634	349.650 ± 103.363	687.168 ± 282.398	303.341 ± 82.500	7.000 ± 0.874
oxoLDPE C_{10}	10 days - 240 h Cycle 60 °C	6.432 ± 0.298	3.529 ± 0.010	9.607 ± 0.056	21.416 ± 0.126	36.075 ± 0.307	19.493 ± 0.150	2.722 ± 0.008
oxoLDPE C_{15}	15 days - 360 h Cycle 60 °C	5.170 ± 0.240	2.833 ± 0.040	6.695 ± 0.102	13.899 ± 0.584	21.967 ± 1.641	12.766 ± 0.471	2.364 ± 0.002

Table S2. Contact angle (CA) measured for unaged and aged oxo-LDPE samples weathered under varied conditions, the data represent an average value with standard error (n = 8)

**n.**	**Samples**	**Ageing conditions**	**CA ± SE**
**1**	oxoLDPE	Unaged	97.08 ± 0.66
**2**	oxoLDPE UV_{48}	48 h UV 50 °C	89.75 ± 1.44
**3**	oxoLDPE UV_{200}	200 h UV 50 °C	83.03 ± 0.51
**4**	oxoLDPE UV_{400}	400 h UV 50 °C	88.69 ± 1.11
**5**	oxoLDPE $UV_{48}t_{120}$	48 h UV 50 °C then 120 h heat 60 °C	81.47 ± 0.83
**6**	oxoLDPE $UV_{48}t_{240}$	48 h UV 50 °C then 240 h heat 60 °C	78.53 ± 1.23
**7**	oxoLDPE $UV_{48}t_{360}$	48 h UV 50 °C then 360 h heat 60 °C	87.17 ± 1.96
**8**	oxoLDPE C_5	5 days - 120 h Cycle 60 °C	89.23 ± 0.57
**9**	oxoLDPE C_{10}	10 days - 240 h Cycle 60 °C	79.01 ± 1.47
**10**	oxoLDPE C_{15}	15 days - 360 h Cycle 60 °C	75.21 ± 0.74

Contact Angle Measurements

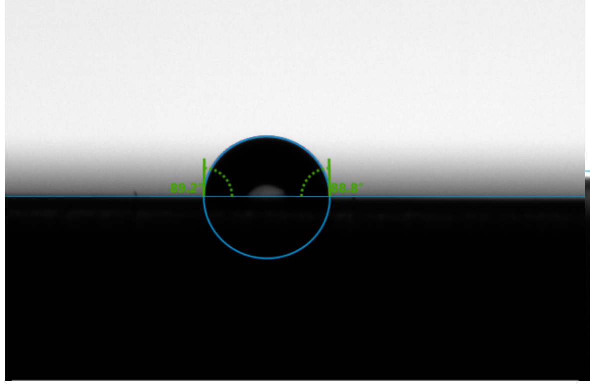

(a) oxoLDPE unaged

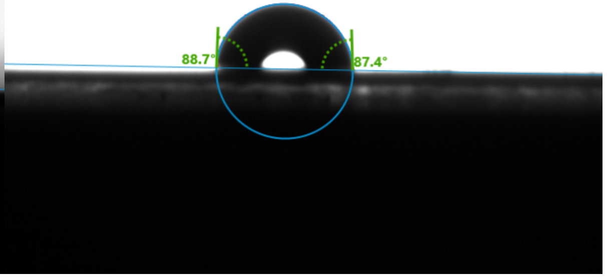

(b) oxoLDPE UV_{48}

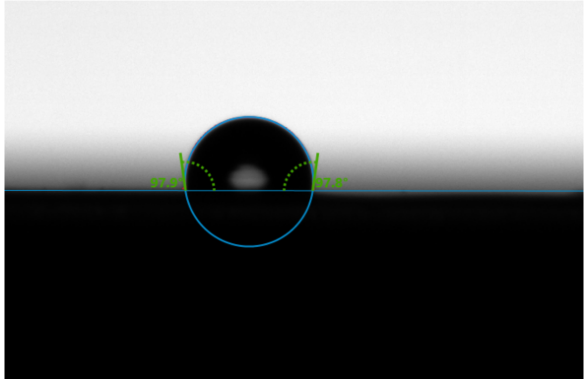

(c) oxoLDPE UV_{200}

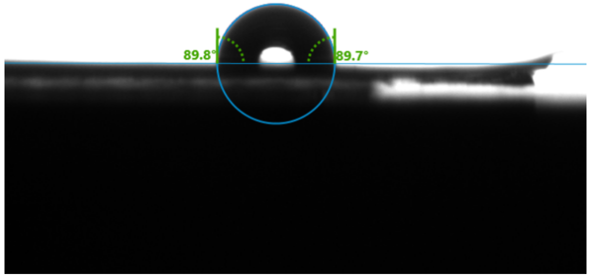

(d) oxoLDPE UV_{400}

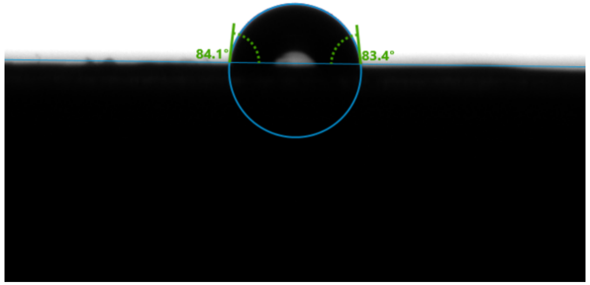

(e) oxoLDPE $UV_{48}t_{120}$

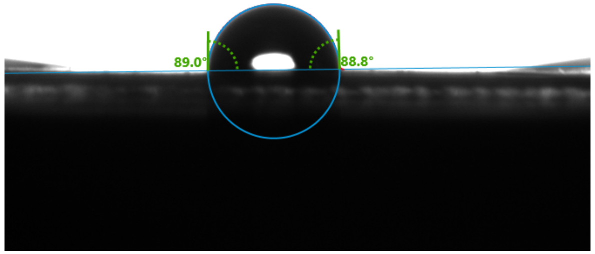

(f) oxoLDPE $UV_{48}t_{240}$

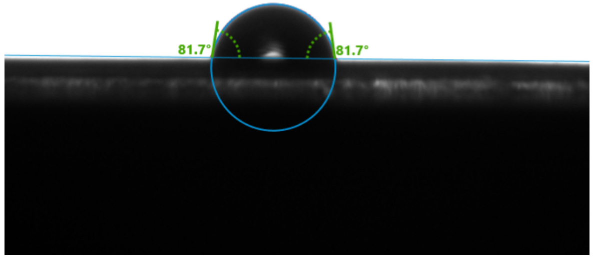

(g) oxoLDPE $UV_{48}t_{360}$

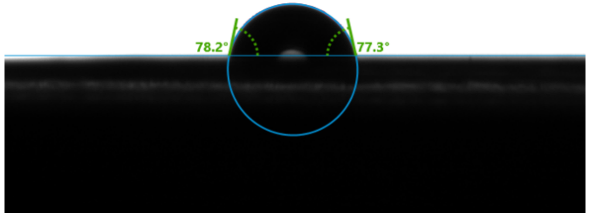

(h) oxoLDPE C_5 – 120h

(i) oxoLDPE C_{10} – 240h

(j) oxoLDPE C_{15} – 360h

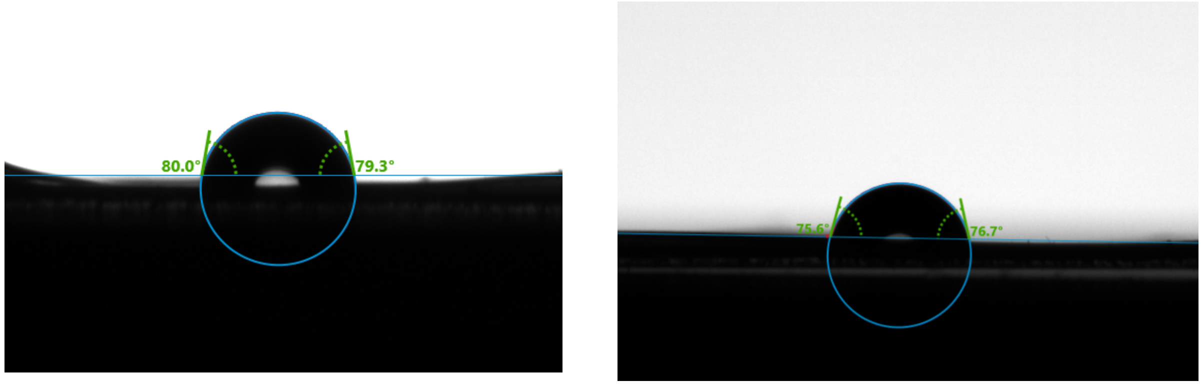

Figure S5. Contact angle measurements (°) for oxoLDPE unaged (a) and aged under UV irradiation 50 °C for 48 h (b), 200 h (c), and 400 h (d), under UV irradiation for 48 h followed by heat only 60 °C for 120 h (e), 240 h (f) and 360 h (g), and with cycle 60 °C for 5 days (h), 10 days (i) and 15 days (j).

Table S3. CO_2 production (ppm) from *Rhodococcus rhodochrous* grown alone and on oxoLDPE samples aged under UV irradiation 50 °C for 200 h (UV_{200}) and 400 h (UV_{400}), and with cycle 60 °C for 15 days (C_{15} - 360 h). Average of three replicates ± standard error.

**Samples**	**Ageing**	**Day 3 CO_2 (ppm) ± se**	**Day 9 CO_2 (ppm) ± se**	**Day 23 CO_2 (ppm) ± se**
R. rhodochrous	N/A	1172.072 ± 15.911	1317.607 ± 18.175	1539.884 ± 34.914
oxoLDPE UV_{200}	200 h UV 50 °C	1331.530 ± 27.340	1568.530 ± 34.771	1802.410 ± 40.239
oxoLDPE UV_{400}	400 h UV 50 °C	1426.697 ± 9.379	1692.547 ± 74.464	1955.962 ± 131.845
oxoLDPE C_{15}	15 days - 360 h Cycle 60 °C	1530.062 ± 119.554	1875.671 ± 114.763	2198.128 ± 88.285
